# A power analysis framework to aid the design of robust semi-field vector control experiments

**DOI:** 10.1186/s12936-025-05454-y

**Published:** 2025-07-18

**Authors:** Andrea M. Kipingu, Dickson W. Lwetoijera, Kija R. Ng’habi, Samson S. Kiware, Mafalda Viana, Paul C. D. Johnson

**Affiliations:** 1 School of Biodiversity, One Health and Veterinary Medicine, University of Glasgow, Graham Kerr Building, Glasgow, G12 8QQ UK; 2 Department of Environmental Health & Ecological Sciences, Ifakara Health Institute, P.O. Box 78 373, Dar Es Salaam, Tanzania; 3 The Pan-African Mosquito Control Association, KEMRI Headquarters, Mbagathi Road, Nairobi, 54840-00200 Kenya; 4 Department of Microbiology and Parasitology, St. Francis University College of Health and Allied Sciences, P. O. Box 175, Ifakara, Tanzania

**Keywords:** Statistical power, Malaria, Mosquitoes, Experimental design, Simulation-based, Type I error

## Abstract

**Background:**

Semi-field experiments are an efficient way of assessing the impacts of potential new vector control tools (VCTs) before field trials. However, their design is critically important to ensure their results are unbiased and informative. An essential element of the design of semi-field experiments is power analysis, which empowers researchers to ensure that only designs with adequate statistical power are adopted. In this study, a methodology was developed, and its use was demonstrated in a tutorial, to determine the required number of semi-field chambers, sampling frequency and the number of mosquitoes required to achieve sufficient power for evaluating the impact of a single VCT or two in combination.

**Methods:**

By analysing data simulated from a generalized linear mixed-effects model, power was estimated for various experimental designs, including short- (24 h) vs. long-term (3 months) experiments and single vs. combined application of interventions (e.g., insecticide-treated nets combined with pyriproxyfen autodissemination).

**Results:**

Although power increased with increasing number of chambers, sampling frequency and the number of mosquitoes, the number of chambers and variance between chambers were the dominant factors determining power relative to all other design choices. High variance between chambers decreased power, highlighting the importance of making conditions similar among chambers, by reducing variation if possible and by rotating variables if not**.** As compared to a single intervention, an additional intervention required an increase in the number of chambers, while short and long experiments were similar in terms of key aspects such as the number of chambers per treatment.

**Conclusion:**

Determining the most efficient experimental design for a semi-field experiment will depend on a balance of design choices and resource constraints. The power analysis framework and tutorial provided here can aid in the robust design of these widely used experiments and ultimately facilitate the development of new vector control tools (VCTs).

**Supplementary Information:**

The online version contains supplementary material available at 10.1186/s12936-025-05454-y.

## Background

Vector control remains one of the most efficient strategies against malaria. Widespread vector control tools, such as insecticide-treated nets (ITN) and indoor residual spraying (IRS), were major factors in the decline in malaria cases, responsible for 68% and 13% reductions in cases, respectively, across sub-Saharan Africa from 2000 to 2015 [1]. However, due to changes in human behaviour [2], the development of mosquito behavioural and insecticide resistance [3–5], and changing mosquito species composition [6–9], these interventions have not been sufficient to continue or improve the trend of declining malaria cases, so they need to be complemented with other tools [10–13].

**Fig. 1:**
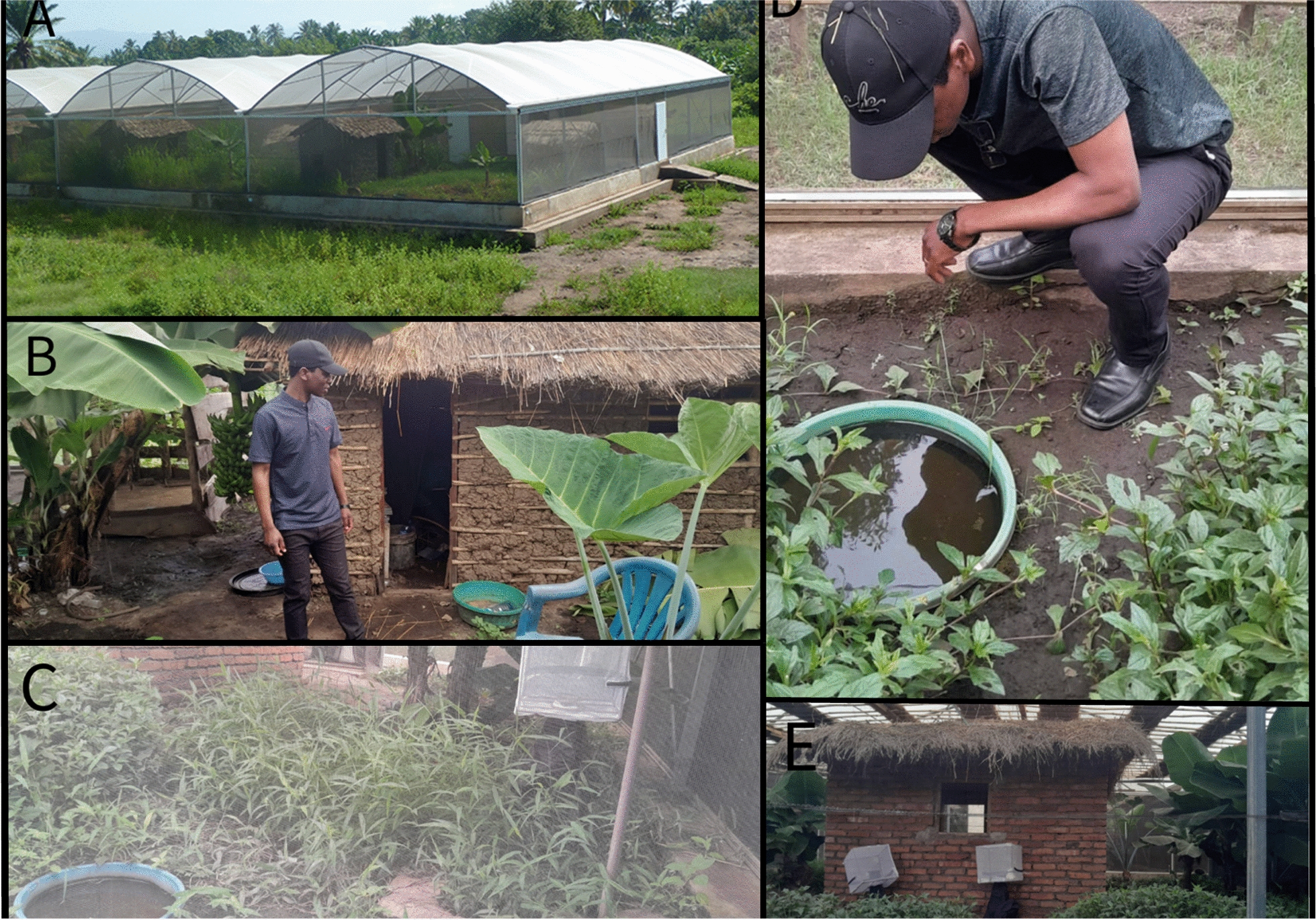
Picture of a semi-field system at the Ifakara Health Institute (Tanzania) with (**A**) the replicated outdoor environment separated by nets keeping mosquitoes inside. Inside the semi-field system, there are the number of (**B**) chambers within which there are (**C**, **D**) artificial larval habitats and vegetation and (**E**) emergence traps for monitoring emerging mosquitoes

New interventions are initially tested in the laboratory for safety and efficacy before moving to the field. Laboratory studies are relatively inexpensive but do not provide evidence of how well an intervention works in the field. In contrast, field studies can provide such evidence but generally require a substantial investment of resources such as time, effort and money. Semi-field experiments (SFE) provide a relatively inexpensive bridge between laboratory and field experiments. SFEs for malaria vector control are conducted within semi-field systems (SFS) (Fig. 1), which can be self-contained habitats placed within the natural ecosystem of a disease vector. These can range in size and conditions and can contain all the requisites for the completion of the vector life cycle [14–18] or be clean rooms with bare grounds [19–25]. Additionally, SFS can be partitioned into compartments (hereafter ‘chambers’) into which mosquitoes are released or emerge and from which they are recaptured or sampled following exposure to the intervention being trialled. Because of their size, the number of chambers available in a SFS typically ranges between 2 to 16 (Fig. 1).

SFE generally adopt one of two extremes of experimental designs: short-term, lasting 24 to 48 h to investigate immediate effects on mosquito mortality; or long-term, > 3 months to investigate dynamic effects that develop over several generations of the mosquito population. For example, interventions that are expected to have a large and fast impact on mortality such as pyrethroid or chlorfenapyr-based ITNs [26–30], attractive targeted sugar baits [19, 23, 31, 32] or spatial repellents [21, 33] could be tested as a short-term intervention in SFS, while those with slow or delayed impacts such as pyriproxyfen (an insect growth regulator typically used to control immature mosquitoes from growing into adults) [34–36] or entomopathogenic fungi [37–39] might be better suited for long-term experiments. In certain SFE studies, adult mosquitoes can be recaptured alive using methods such as light traps or Human Landing Catches. In these cases, the response variable is repellence, as the mosquitoes are alive while being repelled by the interventions. Other SFE studies may focus on counting dead mosquitoes, making mortality the response variable instead. An additional important aspect of any experimental design is selecting designs that give adequate power [40, 41]. Power refers to the probability of identifying a particular effect in a research study, assuming it exists. In the case of SFE, design choices can mean the number of chambers, duration of the experiment, and number of mosquitoes to be recaptured or sampled [42]. The ability to compare power between different designs allows researchers to make informed decisions about which of these designs is more likely to allow them to detect an effect of interest.

Standard analytical power analysis methods available from software packages often only deal with simple analyses such as t-tests, ANOVA or chi-squared tests [43, 44]. Unfortunately, these are not suitable for analysing semi-field experiments as they generally do not allow count data outcomes (e.g. number of mosquitoes) and multiple levels of random variation (e.g., variation among observation within chambers or variation between chambers), both of which are common in SFE. There is a need for a power analysis framework that reflects how the resultant data from SFE will be analysed. There has been no systematic review to date of the use of power analysis in SFE. For illustration, articles in Malaria Journal and Parasites & Vectors were surveyed using the search terms “semi-field” and “mesocosm”. A total of 38 articles (19 from Malaria Journal and 19 from Parasites & Vectors) published between 2020 to 2023 that comprise SFE studies (n = 25) or hut trials (n = 13) which would have been eligible for power analysis were selected (see Additional file 1).

Furthermore, the review has also shown that data from SFEs are usually analysed using generalized linear models (GLMs; 26% of articles reviewed) or generalized linear mixed-effects models (GLMMs; 45% of articles reviewed). Therefore, a power analysis method that also incorporates a similar degree of complexity and flexibility as GLMs or GLMMs will give the most realistic power estimates. When conducting power analysis for GLMMs, it is usual to use an approach called Monte Carlo simulation because of the flexibility and accuracy it gives relative to analytical power analysis methods. Simulations are used to generate multiple datasets that are as similar as possible to the expected datasets resulting from the planned experiments. All simulated datasets are analysed, and the proportion of these datasets giving a significant result is considered the probability of detecting an effect, i.e., the estimate of power. The use of simulation from GLMMs overcomes the limitations of traditional power analysis methods as GLMMs can account for multiple sources of random variation [45]. The advantages of using simulation-based power analysis for overcoming the limitations of analytical methods are well-known and have been illustrated in several studies [45–48]. However, these methods are little used in the design of vector control trials, specifically for SFE.

From the review, eleven (29%) of the selected articles used power analysis to justify the sample size (five from Malaria Journal and six from Parasites & Vectors), of which six shared the same author. Of the eleven articles that used power analysis, 5 articles used the simulation-based power analysis method, 1 article utilized a method for comparing proportions and 6 articles did not specify the power analysis methods used. Although this is a small sample, it suggests that power analysis is underutilized in vector control SFEs. This neglect may in part be attributed to the limitations associated with traditional power analysis approaches or lack of expertise. This study aims to address both of these obstacles by developing a power analysis framework for the design of SFE and providing step-by-step instructions on how to apply it.

To illustrate the value of power analysis to SFE design, guidance and tools are presented through reproducible examples that illustrate the trialling of two malaria vector control interventions, applied independently or in combination (i.e., single and combined) under two experimental scenarios (short-term with static effects and long-term with dynamic effects). This study aims to empower researchers conducting SFEs to estimate power across a range of experimental design scenarios. This knowledge of statistical power, in combination with knowledge of the costs and resource constraints specific to each design, will allow researchers to make informed choices between competing designs. Specifically, the objective is to develop a power analysis framework that can be used to explore the impact on power of varying (i) the number of chambers (i.e., SFS compartments) per treatment, (ii) the number of mosquitoes to be recaptured in the control chamber (for short-term SFEs, recaptured mosquitoes can be used as a proxy of how many mosquitoes should be released), or to be sampled after they emerge from a self-propagating population (for long-term SFEs), (iii) the frequency of sampling mosquitoes, (iv) the amount of variation between chambers, and (v) the chosen size of the intervention effect that can be detected (targeted effect size).

## Methods

### Overview of experimental design choices

It is assumed here that the aim of a vector control experiment is to identify whether the single intervention is better than the control, or if the combined intervention (i.e., there is an interaction effect) is better than expected based on combining (multiplying) the separate effects (i.e., there is no interaction effect). Here, two typical SFE design scenarios, trialling single (i.e., ITN alone) and combined (i.e., ITN and pyriproxyfen autodissemination) interventions were explored. Pyriproxyfen autodissemination (PPFa) is mosquito-assisted larviciding where adult female mosquitoes transfer pyriproxyfen to their aquatic habitats [35]. Although the power analysis framework developed here incorporates two malaria vector control tools (VCTs), namely ITN and PPFa, in practice, these could be any VCTs with similar characteristics. Additionally, two experiment durations were studied: short-term i.e., lasting 24 to 48 h, which intends to investigate immediate effects on mosquito mortality; and long-term, i.e. > 3 months, which intends to investigate dynamic effects that develop over several generations of the mosquito population. For all the scenarios, different SFE designs were tried as follows. First, a varying number of chambers, i.e. 2, 4, 6, and 8 per treatment were used. Second, for frequency of sampling, the short-term experiment had a single sampling point at 24 h but for the long-term experiment (i.e., an experiment taking 3 months) different frequencies were explored namely, monthly sampling consisting of 3 sampling points, fortnightly sampling consisting of 6 sampling points, weekly sampling consisting of 12 sampling points and daily sampling consisting of 90 sampling points. Third, different mosquito abundances recaptured in control chambers were set as 5, 10, 20, 30, 40, 50, 60, 70, 80, 90 and 100 mosquitoes for the short-term experiments while 1, 5, 10, 20 and 40 mosquitoes were used for the long-term experiments. Throughout this paper, recaptured mosquitoes (or mosquitoes to be recaptured) will be specifically used as a proxy of how many mosquitoes need to be released in the control chamber for a short-term SFE.

**Fig. 2:**
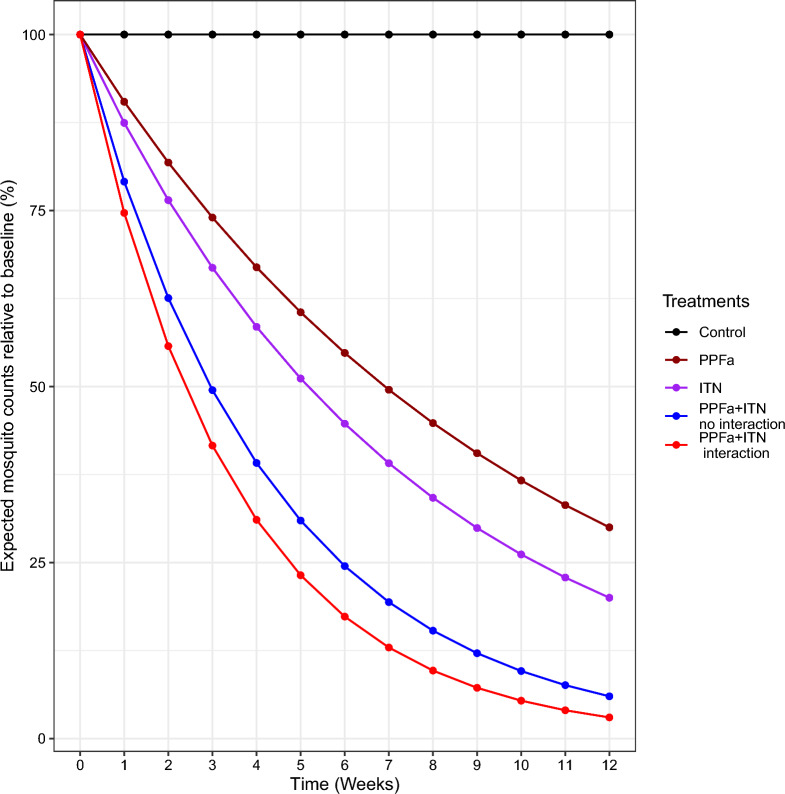
Change in expected mosquito counts over 3 months (12 weeks), comparing the use of ITN and PPFa interventions in a semi-field experiment, showing how much faster the number of mosquitoes is reduced in the chambers combining both ITN and PPFa with (red) and without interaction (blue) than would be expected based on the single effects of PPFa (dark red) and ITN (purple) or no intervention (black)

In this power analysis framework, the target effect size for each intervention was defined as the proportion of mosquitoes remaining at the end of the experiment. E.g., for single interventions, assuming that ITNs result in an 80% reduction in mosquito populations and PPFa results in a 70% reduction, then 20% and 30% of mosquitoes will remain at the end of the experiment (Fig. 2, purple and brown lines), respectively. For combined interventions, where ITNs and PPFa are implemented simultaneously but without interactions, the product of the final proportions, which results in 20% × 30% = 6% of mosquitoes remaining at the end of the experiment (Fig. 2, blue line) was assumed. Finally, for combined interventions with interaction, an interaction effect of 50%, i.e. the 6% of mosquitoes remaining when no interaction occurs will be reduced to 3% when there is an interaction (Fig. 2, red line) was assumed. The example effect sizes given above for ITNs and PPFa are quite large; therefore, the power of the experimental designs to detect four smaller effect sizes and their combinations was explored. All scenarios and designs are summarized in Table 1. Here, zero intervention effect size (0% reduction in mosquito population) represents the null hypothesis. The purpose of including the 0% scenarios in the study was to compare the estimated and nominal (i.e., 5% power) type I rates.

**Table 1:** Experimental design scenarios, study variables and simulated values for short- and long-term experiments

Experimental scenarios	Study variables	R object names	Simulated values
Short-term experiment for single and combined interventions	Number of chambers per treatment	n.ch.per.trt	2,4,6, 8
	Expected number of mosquitoes recaptured in the control group	lambda	5, 10, 20, 30, 40, 50, 60, 70, 80, 90, 100
	Target effect sizes for single intervention (as % reduction)	itn.effect, ppf.effect	0%, 40%, 50%, 60%, 80%
	Target interaction effect sizes for combined interventions (as % reduction in mosquito population)	ixn.effect	0%, 40%, 50%, 60%, 80%
	Inter-chamber variance,${\sigma }_{c}^{2}$	chamber.var	0.0904, 0.1807, 0.3614, 0.9035
	Experimental time period	–	24 or 48 h
Long-term experiment for single and combined interventions	Chambers per treatment	n.ch.per.trt	2, 4, 6, 8
	Sampling frequency (sampling points)	sampl.freq	Daily (90), weekly (12), bi-weekly (6), monthly (3)
	Expected number of mosquitoes to be sampled	lambda	1, 5, 10, 20, 40
	Target effect size for a single intervention	itn.time, ppf.time	0%, 40%, 50%, 60%, 80%
	Target interaction effect size for combined intervention	ixn.ppf.itn.time	0%, 40%, 50%, 60%, 80%
	Time variance,${\sigma }_{T}^{2}$	time.var	0.2266
	Inter-chamber variance,${\sigma }_{c}^{2}$	chamber.var	0.0904, 0.1807, 0.3614, 0.9035
	Experimental time period	–	12 weeks (i.e., 3 months)
	Dispersion parameter of the negative binomial distribution,$\theta$	theta	10

An estimated variance (EV) between chambers of 0.1807 was used in this study, estimated by fitting a negative binomial GLMM to the mesocosm experimental data [49]

Unexplained variation in abundance between chambers (here referred to as inter-chamber variance) is one of the determinants of the statistical power of any SFE study. To quantify the impact of lower and higher inter-chamber variance on statistical power, an estimated variance (EV) of 0.1807 was used based on published SFE data [49] and varied it by factors of 0.5 × , 1 × , 2 × and 5 × (Table 1). This inter-chamber variation could come from various factors including daily heterogeneity, volunteers sleeping beneath the ITNs, recapture methods, location of the chamber and host species.

### Approach for short-term experiments testing single and combined interventions

This section covers the detailed statistical models for short-term SFE testing single and combined interventions where mosquitoes are trapped at a single time point. The number of mosquitoes trapped in the chamber $j$, ${y}_{j}$, were modelled as being drawn from a Poisson distribution, with ${y}_{j}\sim Pois({\lambda }_{j})$. The natural log of expected mosquito counts in the chamber $j,$ is given as

1 $$log\left({\lambda }_{j}\right)={\beta }_{0}+{\beta }_{I}{ITN}_{j}+{c}_{j}.$$

where ${\beta }_{0}$ is the expected log abundance in an average control chamber where neither intervention is present. ${ITN}_{j}$ represents a single ITN intervention that is static over time and is an indicator variable, i.e., 1 in chambers where the intervention is deployed and 0 otherwise. ${\beta }_{I}$ is the coefficient of the covariate ${ITN}_{j}$ and represents the multiplicative intervention effect size on the natural log scale. For example, if an intervention such as an ITN reduces mean vector abundance by 80%, the multiplicative effect is ${e}^{{\beta }_{I}}=0.2$, and ${\beta }_{I}=\text{log}(0.2)$. ${c}_{j}$ is a normally distributed random effect representing variation between chambers that is not explained by the fixed effect of the intervention, such that ${c}_{j}\sim N\left(0,{\sigma }_{c}^{2}\right)$ where ${\sigma }_{c}^{2}$ is an inter-chamber variance. In short-term SFEs, the assumption is that mosquitoes in the control chambers will die naturally or not be recaptured, while those in the treatment chambers will die naturally, not be recaptured or be killed by the insecticides. The decision to use a Poisson distribution instead of a negative binomial distribution (which is simply a Poisson distribution that allows additional observation-level variation, termed overdispersion) in the short-term SFEs was because, with only a single time point available, the variability observed within a single chamber is limited to the between-chambers variance. Often, short-term experiments can be modelled with binomial distributions, where the number of mosquitoes recaptured would be modelled as a proportion of the number released. However, since low recapture rates are common, the number of mosquitoes being sampled becomes unknown (i.e. smaller than the number of mosquitoes released). For this reason, the use of count distributions (Poisson and negative binomial) was adopted here and the Poisson is a good approximation to the binomial at low recapture rates (< 50%), which are common in SFEs [19, 50]; and Poisson GLMMs are considerably faster to fit than binomial GLMMs, which is an important consideration when running multiple simulations. However, recapture rates from some SFEs can be high, exceeding 80% [17, 25, 50, 51], and in these cases, the use of a binomial distribution is recommended. The framework provided here can be easily extended to a binomial distribution, and a previous study [45] also provides a tutorial for simulating from a binomial GLMM in the context of a hut trial, which can provide further support in this expansion.

To test the interaction between two interventions, Eq. 1 was extended to add two predictor terms such that,

2 $$log\left({\lambda }_{j}\right)={\beta }_{0}+{\beta }_{I}{ITN}_{j}+{{\beta }_{P}{PPF}_{j}+{\beta }_{I,P}{ITN}_{j}{PPF}_{j}+c}_{j}$$

where ${\beta }_{I}$ (as in Eq. 1) and ${\beta }_{P}$ are the coefficients representing the effects of the covariates ${ITN}_{j}$ and ${PPF}_{j}$ (the PPF was used here for illustration purposes, although it may not have immediate effect), respectively, which are indicator variables (i.e., 0 or 1) representing the presence in chamber *j* of that specific intervention. ${\beta }_{I,P}$ is a coefficient of the covariate ${ITN}_{j}{PPF}_{j}$ which represents an interaction between ITN and PPFa. Because ${ITN}_{j}{PPF}_{j}$ is the product of the ITN and PPFa indicator variables, it is also an indicator variable, which is 1 only when both interventions are present and 0 otherwise. The interaction coefficient ${\beta }_{I,P}$ indicates how much more rapidly mosquito abundances are reduced by the combined effect of ITN and PPFa compared to what would be expected by combining (multiplying) their individual effects.

### Approach for long-term experiments testing single and combined interventions.

This section provides a detailed model description of a more complex experimental design, a long-term semi-field experiment testing single and combined interventions where mosquitoes are trapped at multiple time points. The number of mosquitoes trapped at a time $i$ in the chamber $j$, ${y}_{i,j}$, was modelled as being drawn from a negative binomial distribution, that is ${y}_{i,j}\sim NB({\lambda }_{i,j},\theta )$. The natural log of the expected number of mosquitoes trapped at a time $i,$ in the chamber $j$, are given as

3 $$log\left({\lambda }_{i,j}\right)={\beta }_{0}+{{\beta }_{T}{t}_{i,j}+ {\beta }_{I}{ITN}_{i,j}+\beta }_{T,I}{t}_{i,j}{ITN}_{i,j}+{\tau }_{i}+{c}_{j}.$$

where ${\beta }_{0}$ is the expected log count in a control chamber at the first time point. ${\beta }_{T,I}$ is the effect of an interaction between time and ITN on mosquito abundance. ${\beta }_{T}$ is the effect of time only on mosquito abundance in control chambers and ${\beta }_{I}$ is the effect of ITN only on mosquito abundance when time is zero. The effects of time and ITN (i.e., ${\beta }_{T}$ and ${\beta }_{I})$ are assumed to be zero in the simulated data (Fig. 2, black and purple colour), this is to help simplify the setting of experimental design choices when simulating the data. However, those effects assumed zero must still be included in the model because in real data it should not be assumed that their effects are zero.

${\tau }_{i}$ and ${c}_{j}$ are normally distributed random effects of time and chamber, respectively, such that ${\tau }_{i}\sim N\left(0,{\sigma }_{\tau }^{2}\right)$ and ${c}_{j}\sim N\left(0,{\sigma }_{c}^{2}\right)$ where ${\sigma }_{\tau }^{2}$ and ${\sigma }_{c}^{2}$ are time and inter-chamber variances, respectively. $\tau$ is incorporated in model (3) because, for a long-term SFE, mosquito abundance is expected to vary randomly between time points.

The $\theta$ is the dispersion parameter of the negative binomial distribution and represents unexplained variation among observations within a single chamber at a single time point. The use of a negative binomial distribution in the long-term SFEs was due to the reason that, when data from multiple time points are available, it is possible to separate consistent variation between chambers from variation between observations within chambers.

To test the interaction between multiple interventions that have dynamic effects over time (see Fig. 2), Eq. 3 was extended to add four predictor terms such that,

4 $$log\left({\lambda }_{i,j}\right)={\beta }_{0}+{{\beta }_{T}{t}_{i,j}+ {\beta }_{I}{ITN}_{i,j}+ {\beta }_{P}{PPF}_{i,j}+\beta }_{T,I}{t}_{i,j}{ITN}_{i,j}+{\beta }_{T,P}{t}_{i,j}{PPF}_{i,j}+{\beta }_{I,P}{ITN}_{i,j}{PPF}_{i,j}+{\beta }_{T,I,P}{t}_{i,j}{{ITN}_{i,j}PPF}_{i,j}+{\tau }_{i}+{c}_{j}.$$

where ${\beta }_{T,I}{t}_{i,j}{ITN}_{i,j}$ corresponds to the change in abundance over time due to ITN relative to changes occurring in the control chambers and ${\beta }_{T,P}{t}_{i,j}{PPF}_{i,j}$ corresponds to the change in abundance over time due to PPFa relative to changes in the control chambers. ${\beta }_{T,I,P}{t}_{i,j}{{ITN}_{i,j}PPF}_{i,j}$ corresponds to the change in the combined treatment chambers relative to what would be expected by combining (multiplying) the effects of change over time from the single treatment chambers. ${\beta }_{T}$ is the effect of time only in the control chamber, ${\beta }_{I}$ is the effect of ITN only and ${\beta }_{P}$ is the effect of PPFa only on mosquito abundance. ${\beta }_{T,I}$ and ${\beta }_{T,P}$ are the effects of the interaction between time and ITN or time and PPFa on mosquito abundance, respectively, while ${\beta }_{I,P}$ is the effect of the interaction between ITN and PPFa only on mosquito abundance. The effects of time, ITN, PPFa and ITNxPPF (i.e., ${\beta }_{T}$, ${\beta }_{I}$, ${\beta }_{P}$, and ${\beta }_{I,P}$) are assumed to be zero in the simulated data (Fig. 2), however, they must still be included in the model because in real data they should not be assumed that their effects are zero. ${\beta }_{T,I,P}$ is the effect of the interaction between time, ITN and PPFa on mosquito abundance.

### Power estimation

Based on Eqs. 1, 2, 3, 4 and the parameter values in Table 1, 1000 data sets for each experimental scenario were simulated. The simulated datasets were analysed by fitting the generalized linear mixed-effect models (GLMMs) from which they were simulated and testing for the intervention effect using the *glmer* function of the *lme4* R package [52], which fits GLMMs using maximum likelihood. Power was estimated as the proportion of simulated datasets in which the null hypothesis of no intervention effect was rejected using a significance threshold of p < 0.05, where the p-value was calculated using Wald z-tests from the GLMMs. Sufficient power was defined as an estimated power of at least 80%. The number of simulated datasets of 1000 was chosen as a trade-off between adequate precision and feasible computation time (1000 data sets give a margin of error of ± 2.5% when true power is 80%).

### Tutorial—practical application

In this section, an R tutorial to illustrate a step-by-step estimation of the statistical power of a short-term SFE with a single intervention is provided. This tutorial is provided to empower researchers with little or no expertise in using a simulation-based power analysis for SFE. This tutorial covers a simple scenario only; however, one can expand this to accommodate more complex SFE scenarios, such as additional interventions or long experiments with single or combined interventions. (see doi: 10.5281/zenodo.11186503) [53].

In sub-sections (i)-(iv) below, simulation and power analysis methods for a single data set are illustrated. Sub-sections (v)-(vii) show how to simulate multiple datasets and estimate power based on experimental scenarios and parameter choices in Table 1. The data sets were simulated under the assumption that the alternative hypothesis (H1) is true, i.e., the intervention effect is not zero (excluding scenarios when 0% effect was simulated). Some of the parameters e.g., *n.ch.per.trt* and *lambda* (Table 1) were chosen based on prior knowledge/experience and per communication with other scientists. Other parameters e.g., *chamber.var,* were estimated using the data from the previous SFE in Ng’habi et al*.* [49].

The total number of chambers per treatment, *n.ch.per.trt,* indicates how many chambers known as “replicates” are present per treatment. The number of chambers can also mean the same chamber for multiple SFE rounds. In the R code below, varying SFE design choices were set and simulated either single or multiple data sets based on the assigned design choices followed by fitting the simulated data to the GLMM. After GLMM fitting, power estimation is performed by calculating a proportion of the simulated data set whose p-values are less than 0.05. The tutorial was implemented in R version 4.2.3 [54] using multiple libraries including *ggplot2* [55] for producing plots, *lme4* for fitting GLMMs [52], and *dplyr* for data manipulations [56].

(i) The setting of experimental design scenarios and parameter choices in R

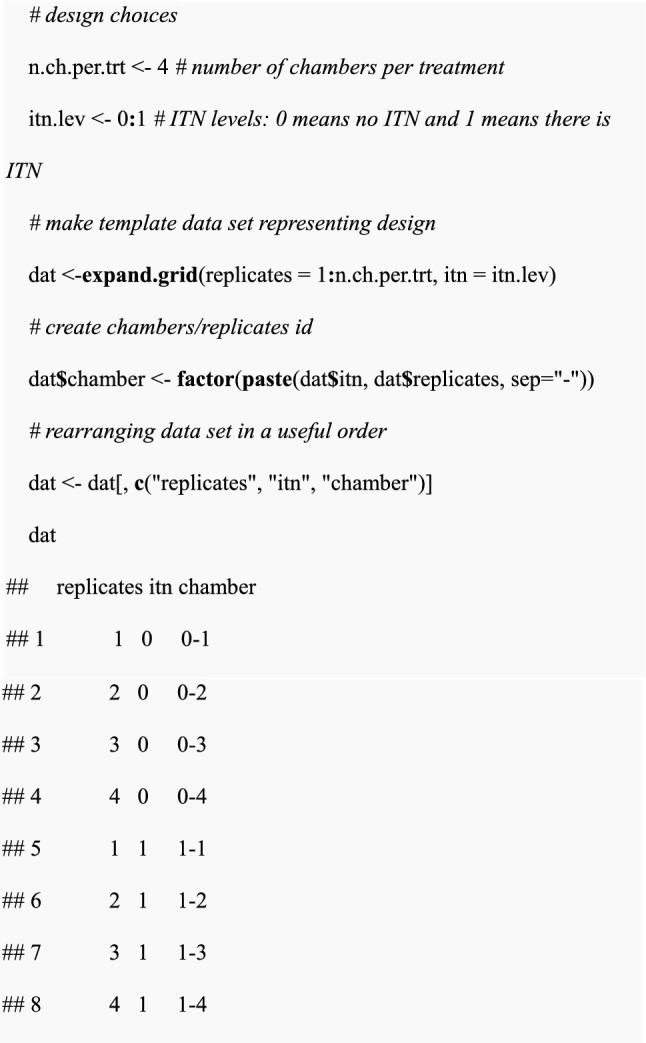


Assign values for all parameters for fixed and random effects:
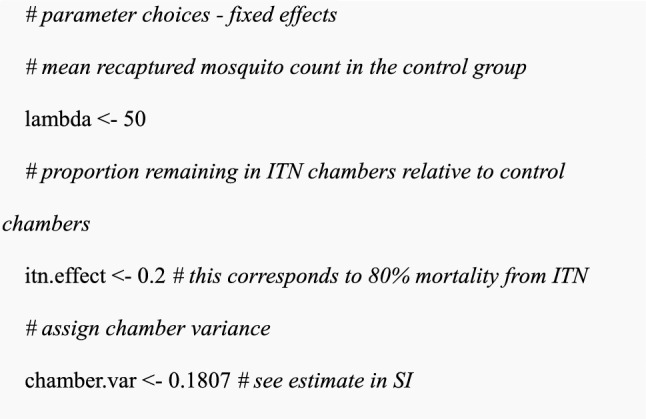


Calculate additive coefficients for fixed effect parameters by applying the log link function to the multiplicative effect of an intervention (i.e., log(0.2) for ITN).
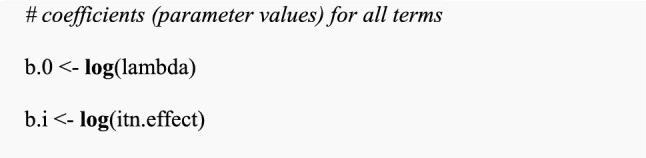

(ii) Simulation of a single data set

After setting experimental design scenarios and parameter choices, the inter-chamber variance, a single data set can now be simulated. Using the simulated linear predictor (sum of terms which include coefficients e.g., ${\beta }_{0}$ as an intercept and ${\beta }_{I}$ as a slope with their associated explanatory variables), expected mosquito counts will then be generated using a Poisson distribution.
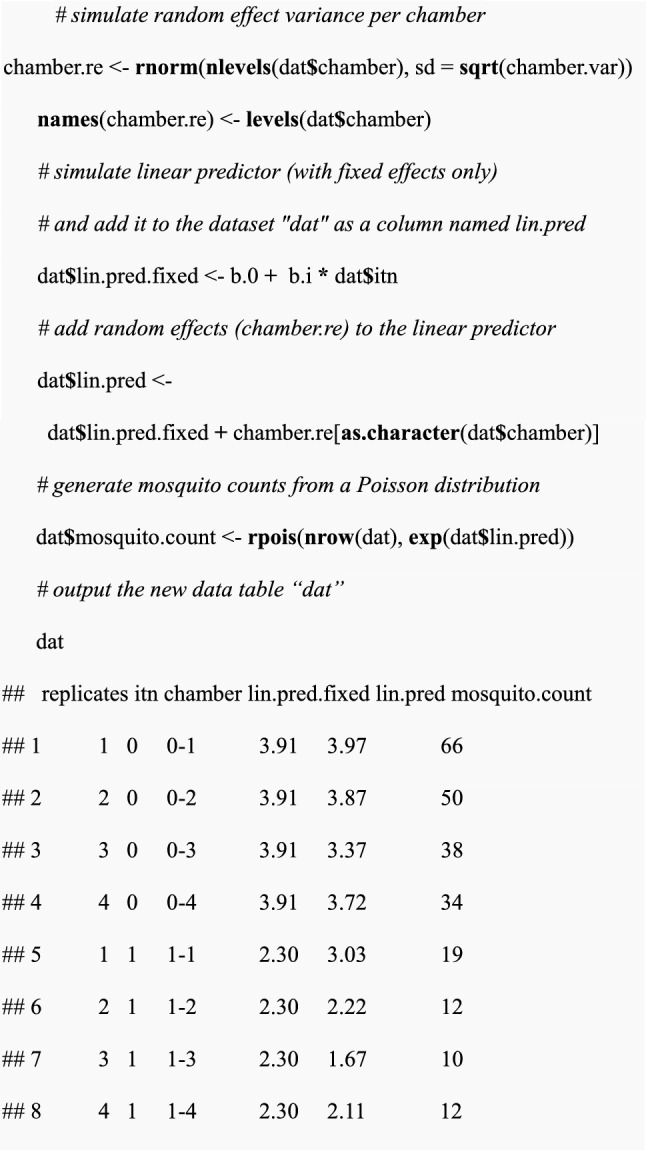

(iii) Perform a statistical test for a simulated data set to calculate the p-value

Fitting the simulated data in “dat” to the GLMM model using the *glmer* function. The model named “model.itn “ will incorporate a response variable which is the expected mosquito counts denoted by “mosquito.count”, a fixed effect for a single intervention (i.e., ITN alone) and a random effect between chambers. From the model summary, the p-value is extracted using the *coef()* function.
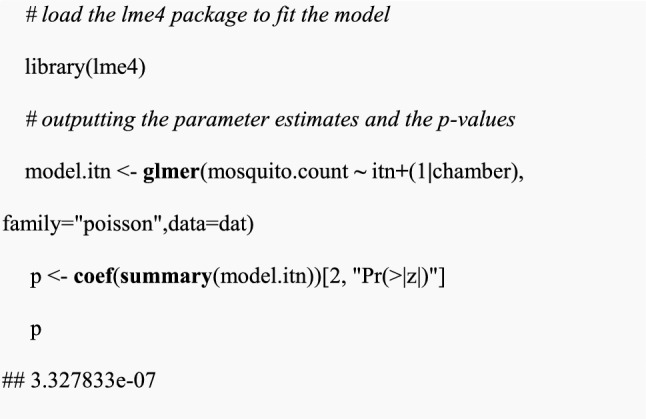


In this example, the p-value was 3.3 × 10^–7^, which is less than 0.05, therefore a significant intervention effect was detected.

(iv) Power estimation for a single data set

In sub-sections (i-iii), an illustration of how to simulate and calculate a p-value for a single data was provided. Since power cannot be estimated from a single simulation, there is a need to simulate multiple data sets as shown in subsequent sections (v-vii).

(v) Simulation of the multiple data sets

A function called “sim.dat.fun” was created to automate the simulation process described in sub-section (ii) above. The "sim.dat.fun" function takes a design table “dat”, coefficients “b.0” and “b.i” and inter-chamber variance “chamber.var” as input parameters. The function then produces a table “dat” containing mosquito counts as an output.
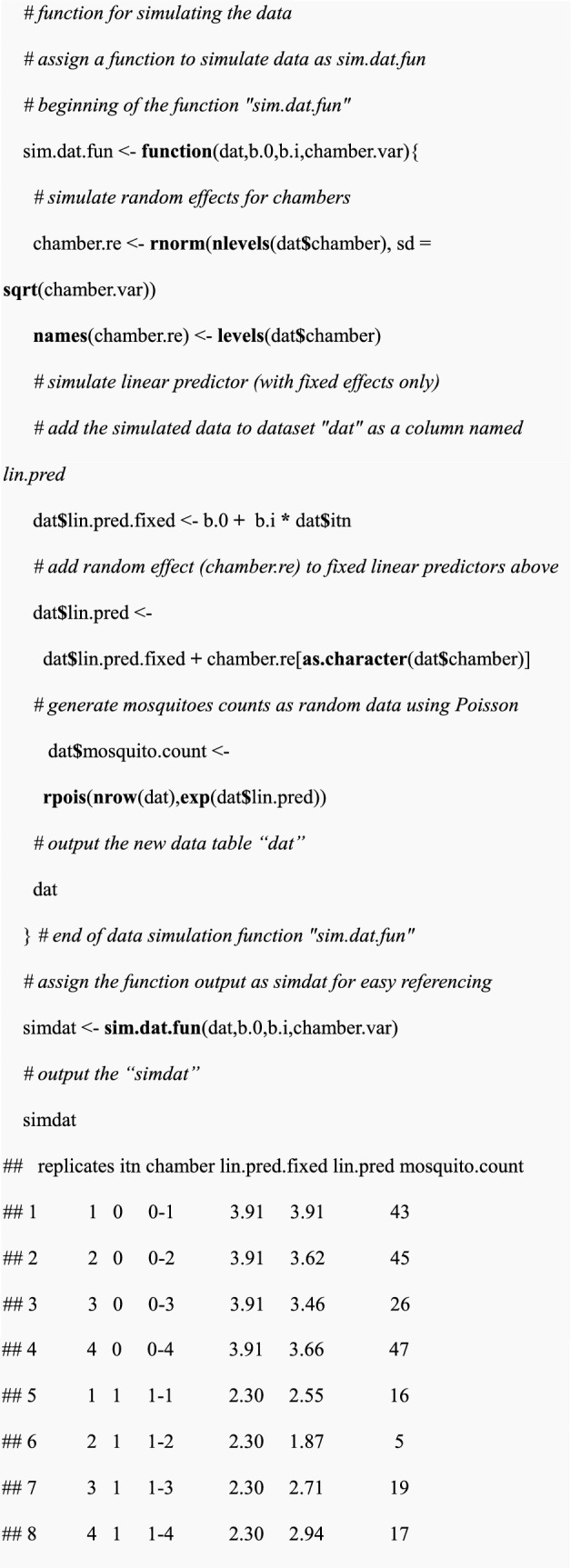

(vi) Perform a statistical test for the simulated data sets to extract p-values

In this case, a function called “sim.mos.pval” was created to automate the function “sim.dat.fun” and outputs p-values. The function “sim.mos.pval” will output the p-values from the GLMM model with a Poisson distribution. The fitted model denoted by “model.itn” will incorporate a response variable which is the expected mosquito counts denoted by “mosquito.count”, fixed effects which are ITN, and random effect between chambers.
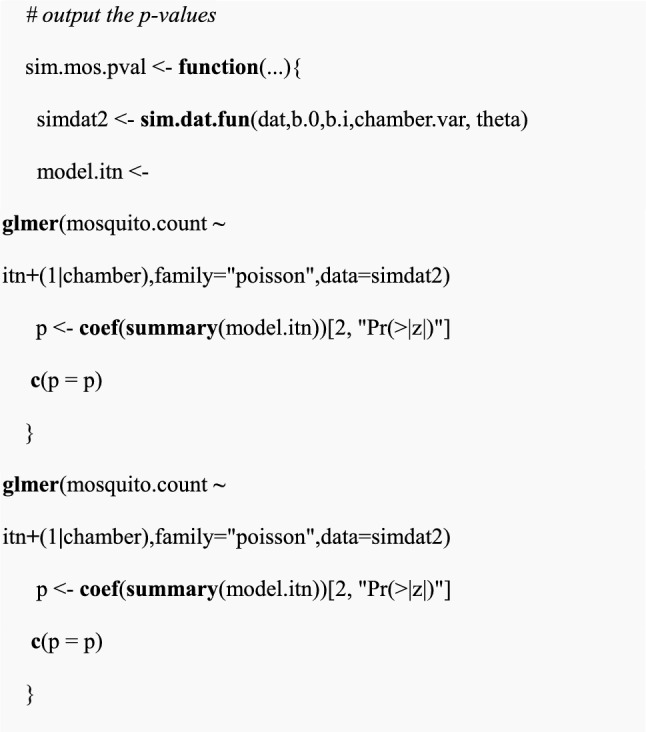

(vii) Power estimation for multiple data sets

Here, a function “sim.pvals.list” was created to output power estimate by updating function “sim.mos.pval” multiple times based on the number of simulations “nsim” provided. Again, another function called “sim.pvals.list” was created to output a list of p-values by automating the function “sim.mos.pval” to simulate multiple data sets based on the number of simulations “nsim” assigned. Therefore, the percentage of the data sets whose p-values are less than 0.05 is the power estimate.
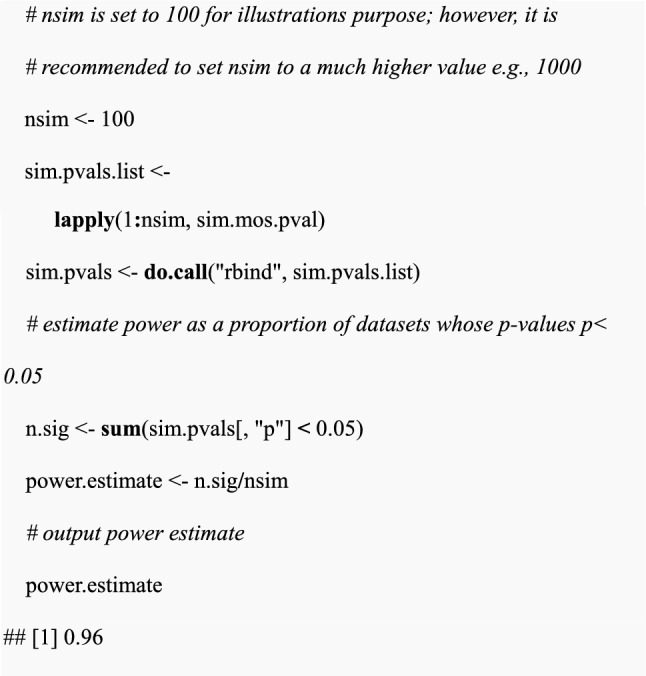


For the simulations reported in the results sections, the power analysis methods illustrated in the tutorial above were used and expanded to estimate power for all combinations of scenarios, short- vs. long-term SFE and single vs. combined interventions. To promote reproducibility, the R code used to produce the results has been made freely available on an online repository (see doi: 10.5281/zenodo.11186503) [53].

### Type I error rate

**Fig. 3:**
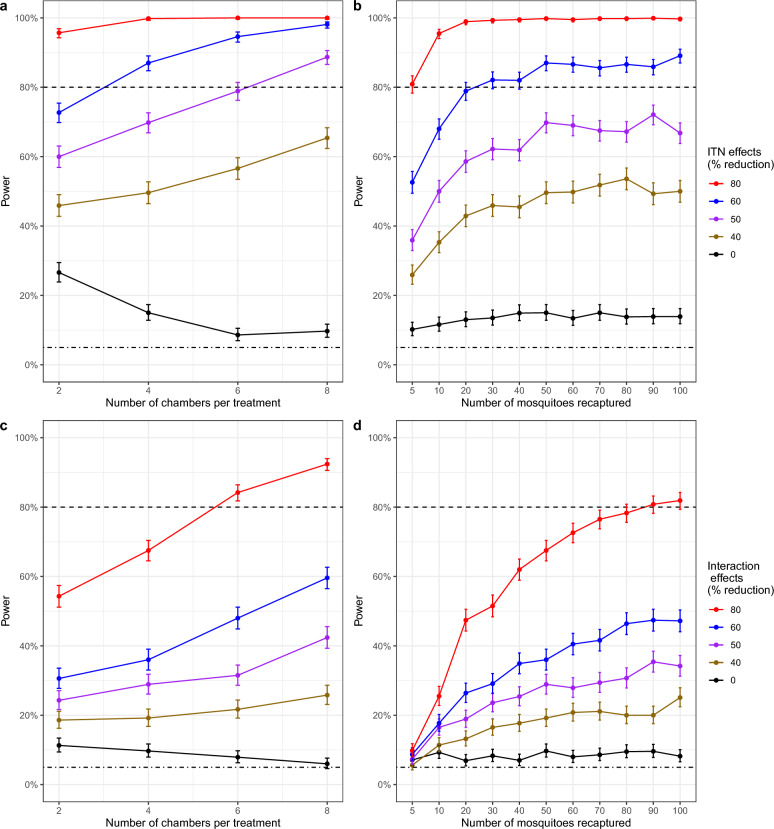
Statistical power obtained from different short-term SFE designs. Top panels (**a** and **b**) show the power expected when testing single interventions (ITN) and bottom panels when testing combined interventions (ITN and PPFa; **c** and **d**) with increasing (**a** and **c**) number of chambers per treatment or (**b** and **d**) number of mosquitoes recaptured. Different coloured lines correspond to varying effects or interaction sizes, i.e., % reduction in mosquito population. The dashed line is 80% power, and the dot-dashed shows a 5% power which is a type I error rate, which is the expected power when the effect size is zero. Error bars show 95% confidence intervals. Estimated variance (EV) was used in both (**a**-**d**) while 50 mosquitoes were used in (**a**) and (**c**), and 4 chambers per treatment in (**b**) and (**d**)

**Fig. 4:**
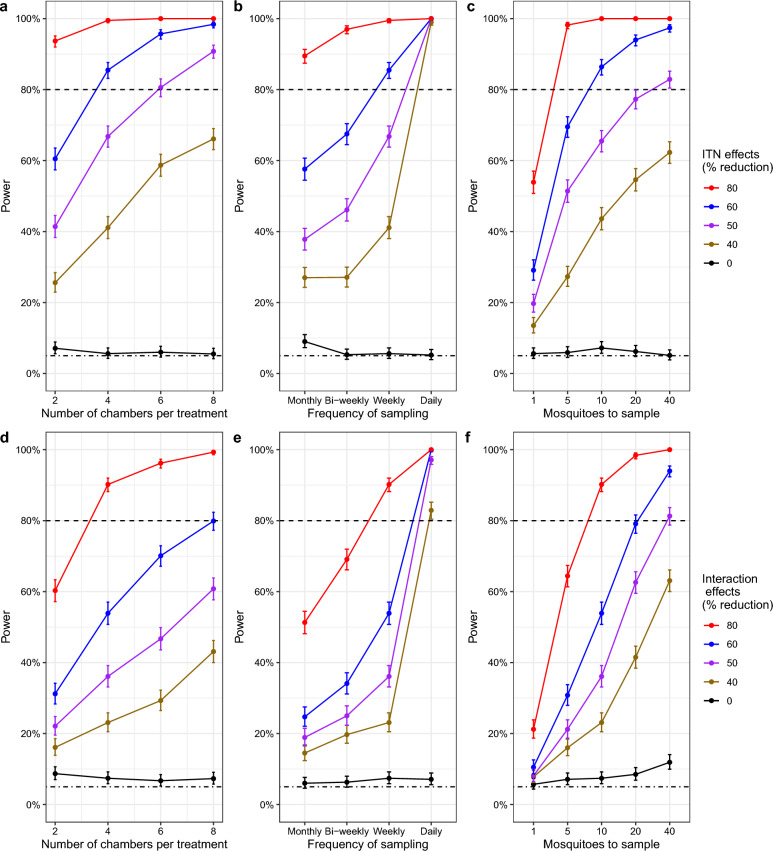
Statistical power obtained from different long-term SFE designs. Top panels (**a**, **b** and **c**) show the power expected when testing single interventions (ITN) and bottom panels when testing combined interventions (ITN and PPFa; **d**, **e** and **f**) with increasing (**a** and **d**) number of chambers per treatment or (**b** and **e**) frequency of sampling or (**c** and **f**) number of mosquitoes to sample. Different coloured lines correspond to varying effects or intervention sizes, i.e., % reduction in mosquito population. The dashed line is 80% power and the dot-dashed shows 5% power which is a type I error rate, which is the expected power when the effect size is zero. Error bars show 95% confidence intervals. Estimated variance (EV) was used in both (**a**-**f**). A total of 10 mosquitoes and weekly sampling were used in (**a**) and (**d**), four chambers per treatment and 10 mosquitoes in (**b**) and (**e**), and four chambers per treatment and weekly sampling in (**c**) and (**f**). The main effects used for ITN and PPFa in (**d**-**f**) are 80% and 70%, respectively

Before reporting power estimates using GLMMs, it is recommended to check whether the estimated type I error rate (i.e. estimated power at zero effect size; Fig. 3, dot-dashed lines) is equal to the nominal type I error rate, which here is 5%. Checking type I error rate estimates is useful for identifying inflated power estimates and more generally for identifying scenarios where GLMMs are unreliable. In this case study, some of the power estimates were inflated, particularly when the number of chambers per treatment was 2. For example, the type I error rate was 15% when there were 2 chambers per treatment for short-term SFEs (Fig. 3 a, c). However, the estimated type I error rates were less severe (slightly inflated) in long-term SFEs at less than 8% (Fig. 4). Type I error rate estimates across all scenarios were averaged at 8%, 3% higher than the nominal type I error rate (Fig. 3 & Fig. 4). Generally, the type I error rate was more inflated in short-term SFEs with scenarios that involve fewer than 6 chambers per treatment than in long-term SFEs testing single and combined interventions.

## Results

### Short-term experiments testing single and combined interventions

Power increased with increasing number of experimental chambers per treatment and the number of mosquitoes to be recaptured from each experimental chamber (Fig. 3). Additionally, the target effect size of the interventions also had a large impact on power in each scenario. Power increased initially with recaptured mosquitoes, then plateaued for single interventions with around 50 mosquitoes, and combined interventions with around 80 mosquitoes (Fig. 3 a and b). Power is 87% at four chambers per treatment, 50 mosquitoes recaptured and 60% reduction in mosquito population (Fig. 3a, blue solid line). A minimum of 10 mosquitoes would need to be recaptured in each treatment chamber to ensure at least 80% power assuming that the only interest is to detect effects at least as large as an 80% reduction in mosquito density (Fig. 3b).

The higher the targeted interaction effect size, the higher the power of the experimental design. However, within target interaction effect sizes (Fig. 3c and d), although there was an increase in power with an increasing number of chambers per treatment: adequate (> 80%) power could only be achieved at the highest interaction effect size (i.e., > 80% reduction) with a minimum of 6 chambers per treatment (Fig. 3c). If a target interaction effect size results in a 60% or less reduction, none of the scenarios was sufficient to give enough power to detect the smallest effect of the intervention (Fig. 3c). With four chambers per treatment, only designs recapturing at least 90 mosquitoes with a target interaction effect size of an 80% reduction in the mosquito population provided adequate power (Fig. 3c). The increase in power above 80% at a minimum of 6 chambers per treatment and high target interaction effect size indicates the necessity for the SFE studies to consider the use of a large number of treatment chambers (at least 4 and preferably 6 chambers) to ensure that the target treatment effects are detected. By comparing the results in Fig. 3, testing a single intervention would require fewer chambers than would be required when testing combined interventions i.e., the increase in the number of interventions increases the number of chambers to be used per treatment. It should also be noted that designs with four or fewer chambers gave the most inflated type I error rates, suggesting that even the low levels of power achieved in these scenarios are likely to be inflated.

### Long-term experiments testing single and combined interventions

Power was higher with more chambers, increased sampling frequency, and a higher number of mosquitoes to be sampled per chamber (Fig. 4). Enough power was achieved at a minimum of 4 chambers per treatment (Fig. 3a), weekly sampling (Fig. 4b), and 10 mosquitoes to be sampled per week (Fig. 4c) with only a 60% reduction in mosquito population by ITN. More than 90% power was achieved at 4 chambers per treatment (Fig. 4d), weekly sampling (Fig. 4e), and 10 mosquitoes to be sampled per week (Fig. 3f) with an 80% reduction in population by ITN and PPFa interaction. The higher the ITN and PPFa interaction effect, the power of the experimental study is higher with the increased number of chambers per treatment, sampling frequency and mosquitoes to be sampled. 100% power was attained at the highest interaction effect (i.e., 80% reduction in mosquito population) by maximizing either the number of chambers, sampling frequency, or the number of mosquitoes recaptured (Fig. 4d-f).

### Inter-chamber variance affects power

**Fig. 5:**
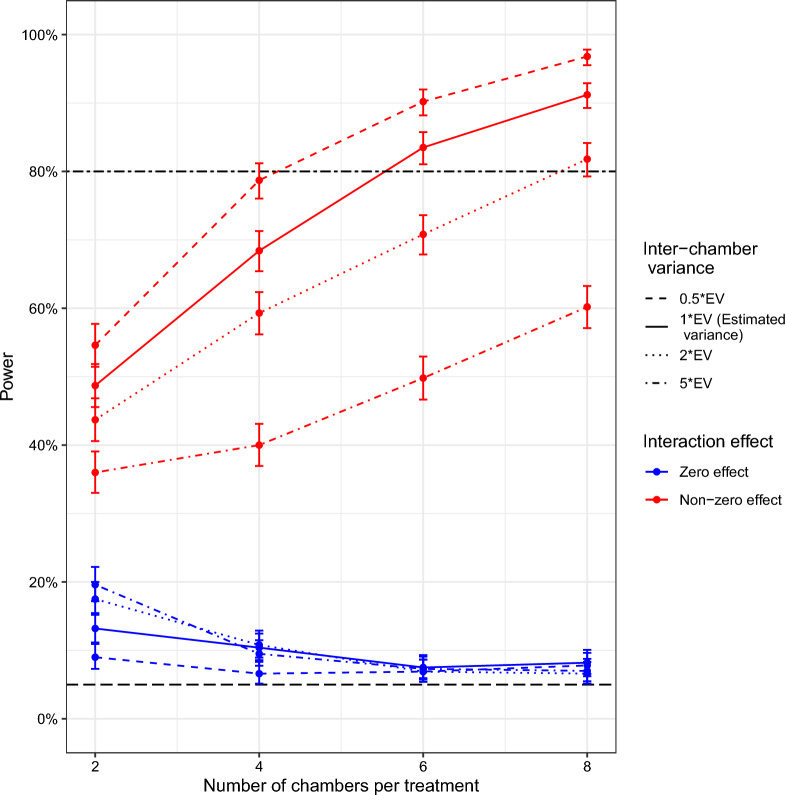
The effect of varying inter-chamber variance on the relationship between power and the number of chambers per treatment. Inter-chamber variance was varied from the estimated variance of 0.1807 (EV, solid line which corresponds to a red line in Fig. 3c) to half (dashed line), double (dotted), and quintuple (dash-dotted line) its original value. Error bars show 95% confidence intervals. The red colour represents the target non-zero interaction effect (80% was used here) and the blue colour type I error rate (0% interaction effect). The two-dashed line is the 80% power, and the long-dashed line (black colour) is the nominal type I error rate of 5%. In this case, a total of 50 recaptured mosquitoes were used and the main effects explored for ITN and PPFa were 80% and 70%, respectively

The estimated type I error rate was 8% (i.e., 3% higher), except for when there are only 2 chambers per treatment for a short-term semi-field experiment (SFE) testing combined interventions, which had a type I error rate > 9%. For the short-term SFE testing both single and combined interventions, the more variation between the treatment chambers the less power there was (Fig. 5, dotted and dash-dotted red lines). In contrast, for the long-term SFE testing of both single and combined interventions, different inter-chamber variances resulted in closely related power estimates. For estimated variance (EV, which corresponds to the red line in Fig. 3c), power was 68.5% when 4 chambers per treatment were used and 50 mosquitoes recaptured, which increased to 84% and 91% at 6 and 8 chambers per treatment, respectively (Fig. 5, solid red line). When the inter-chamber variance was halved to EV/2, 79% power was attained at only 4 chambers per treatment but then increased to 90% and 97% at 6 and 8 chambers per treatment, respectively (Fig. 5, dashed red line). In contrast, when inter-chamber variance was doubled to EV × 2, power was 59.5%, 70.5% and 82% at 4, 6 and 8 chambers per treatment, respectively (Fig. 5, dotted red line). Additionally, a fivefold increase in inter-chamber variance lowered power to below 60% irrespective of the used number of chambers per treatment (Fig. 5, dash-dotted red line).

## Discussion

This study provides guidance and a tool to empower semi-field experiment (SFE) researchers to explore the relationship between design choices and predicted power. In this study, the impact on the power of varying the number of experimental chambers, the number of mosquitoes recaptured in the control (as a proxy for the number released), the frequency of sampling adult mosquitoes, the amount of variation between chambers, and the size of the target intervention effect were investigated.

Before reporting simulation-based power estimates using GLMMs, it is beneficial to check whether the estimated type I error (i.e., false positive) rate is correct. Type I error rates, which are equivalent to estimated power at zero effect size, were inflated (> 5%) for all experimental designs, suggesting that Wald z-tests from GLMMs fitted using maximum likelihood give inflated type I error rates. In this study, type I error rate inflation was particularly severe (> 8%) for designs with fewer chambers per treatment (≤ 4), due to severely biased p-values generated from Wald z-tests. For a single analysis, this inflation problem can be resolved by adjusting p-values, for example using the function *simulateLRT* in the *DHARMa* R package with which performs simulated likelihood ratio tests for GLMMs based on the parametric bootstrap [57, 58]. However, such simulation-based methods are too slow to be feasible as part of simulation-based power analysis. Therefore, in addition to identifying scenarios where power is likely to be over-estimated, estimating the type I error rate can also alert researchers to scenarios that generate potentially unreliable results from GLMMs, without which knowledge they might publish false positive results.

One of the main findings of this study was that for most realistic SFE designs, the power estimate was below the conventional threshold for acceptability of 80%, except for experimental scenarios with an extremely large target effect size. That is to say, for the experimental scenarios covered in this study, current SFEs seem to be underpowered for more realistic effect sizes. It is perhaps one of the general limitations of SFE that, unless a large-scale semi-field system is built with five or ten times as many chambers or, more realistically, repeat the experiment multiple times with rotated (e.g., using a Latin square design) or randomized chambers per treatment (which would effectively double or multiply the number of chambers), it will difficult to be able to detect anything less than very large effects. The power analysis framework presented here is intended to give SFE researchers the tools to decide how large a semi-field system would need to be, or how many repetitions of the experiment would be required, for a particular effect size of a given intervention.

Power was expected to increase with more chambers, more mosquitoes, higher sampling frequency and lower inter-chamber variation, but the relative importance of these factors in a range of realistic SFE scenarios was unknown. In this study, a realistic SFE required a minimum of 4 chambers to detect single (non-interaction) effects but may be underpowered for effects below a 40% reduction. To obtain adequate power and maintain an acceptable type I error rate, a minimum of 4 and preferably 6 chambers per treatment was required to detect higher interaction effects (i.e., ≥ 80% reduction), suggesting that using designs with fewer chambers would have resulted in an underpowered study for lower effects. In general, the results showed that two chambers per treatment are too few and insufficient to ensure the detection of the interaction effect between PPFa and ITN*.* However, several ways may help increase the number of chambers per treatment, that do not involve building a large-scale semi-field system or repeating the experiment multiple times [14, 15, 59]. For example, one may consider whether both single interventions are needed in the same experiment and potentially exclude the negative control.

Human Landing Catch (HLC) is one of the standard methods for sampling adult mosquitoes, however, it demands a lot of effort in terms of time, money, and logistics such as a trained supervisory team and supplies at the collection sites [60]. Other common recapture methods include CDC light traps and prokopack aspirators. These can be used indoors or outdoors and across species (see SFE studies in Additional file 1 for more recapture methods) but will also determine the life history stage of the mosquitoes captured (e.g., fed or blood-seeking). For instance, HLC limits the collection of fed mosquitoes, whereas aspiration methods can capture both fed and unfed mosquitoes. Beyond the recapture method, recapture rates are also influenced by other factors, including the size of the chambers, host species and the setting, from climate to location (indoor vs outdoor) and the amount of vegetation and breeding sites [19]. Consideration of the expected recapture rates and life stage of mosquitoes to be recaptured will help determine the experimental design for the power analysis. While large SFS might not often be used in short-term experiments as it makes recovering the mosquitoes more difficult, such setups do still occur (often in more bare chambers) [17, 19, 61], and this framework is independent of the size or conditions of the SFS. Given the variable recapture rates in these systems [19, 50, 61], and the desire to increase the complexity of the models while keeping the biological framework consistent, recaptured mosquitoes was used as opposed to released mosquitoes.

The selection of sampling frequency involves a balance between statistical power and the amount of resources to be invested in an experimental study, and this study illustrated how this balance point could be found. Although without providing scientific reasons, most studies with SFE consider weekly or bi-weekly sampling of mosquitoes [62]. Results from this study indicated power increases as sampling frequency increases from monthly to daily. If the effect size is sufficiently large, then daily sampling may not be necessary; instead, one could opt for weekly or bi-weekly sampling. If the effect size is low, then in theory, daily sampling would be suitable to improve power, but an increase in population size would be needed to accommodate consistent removals or consider a frequency of sampling somewhere between week and daily or update the power analysis to account for these. Under this study scenarios, bi-weekly sampling did not improve power substantially compared to monthly sampling, although even this small increase could bring borderline designs above 80% power.

It is difficult to obtain enough mosquitoes for use in the SFEs because of the high operational cost of rearing them in insectaries. Additionally, other species such *Anopheles funestus* is even more challenging to maintain in insectary settings, making it hard to establish laboratory colonies [63]. Due to its importance, researchers need to determine the number of mosquitoes they are supposed to use for SFEs, which will provide them with successful and informative experiments. Since there is no standard way to identify the total number of mosquitoes to be released, especially in long-term experiments, understanding the expected number of mosquitoes to be recaptured can be a good proxy to help determine how many mosquitoes should be released to maximize the power of the study. Low release or recapture rates (e.g., recapturing fewer than 80% or 50% of mosquitoes) and using a limited number of chambers (e.g., only two per treatment) are quite common in most of the SFEs [19, 37, 50, 64]; therefore, there is a risk for numerous semi-field studies being underpowered. This study suggest that recapturing 50 mosquitoes in six chambers per treatment achieved adequate power (> 80%) to detect an effect size of 50% reduction in mosquito abundance. Increasing variation between chambers in the mean number of mosquitoes recaptured resulted in lower power in the short-term SFEs and reducing inter-chamber variation increased power. In contrast, for the long-term SFEs, different inter-chamber variances resulted in similar power estimates. It is suggested that conditions should be kept as similar as possible across all treatment chambers, which can be done, for example, by rotating volunteers who capture mosquitoes or hosts for mosquitoes’ blood meals.

While the power analysis methods presented here provide valuable insights into optimizing SFE design, they also have limitations. The framework relies on simulation-based power analysis with GLMM-fitting methods, which have higher computational demands than traditional power analysis methods. On the other hand, this study showed that daily sampling would lead to adequate power; however, this would probably remove the population. This study also focused on repellence, while many entomological experiments assess mortality. The focus of this study was to develop a statistical power analysis framework and produce an R tutorial to assist in the design of robust vector control experiments in semi-field systems. Results for two specific scenarios are shown, but these can be easily adapted and extended to other intervention scenarios including long-term SFE with non-dynamic effects, or effects changing over time. As the framework was adapted from a Poisson to a Negative binomial model for the long-term experiment, other adaptations might be required. For example, a binomial model might be preferred in the presence of high variability in the recaptured mosquitoes (e.g., associated with chamber variance) or high recapture rates as larger numbers of mosquitoes would be expected. Alternatively, a multinomial model could be a good option for complex dependencies between modes of action [65, 66]. In addition, this framework could be used for other widely used experimental systems such as hut trials, where instead of chambers, outdoor experimental huts are used. The uptake of power analysis methods will improve the quality of SFE and as a consequence provide a more robust evaluation of the impacts of new vector control interventions on mosquito populations. Future research directions may include extending the framework to different vector control experimental scenarios or exploring alternative SFS experimental designs. For instance, it will be essential to look separately into the life history stages, such as knowing the number of mosquitoes in various categories such as alive unfed, alive fed, dead unfed and dead fed. Additionally, future work may focus on expanding this work to a web-based application such as an R shiny application and R packages for an interactive power analysis framework.

## Conclusions

Determining the most efficient experimental design for a semi-field experiment (SFE) will depend on a balance of design choices and resource constraints. The power analysis framework and tutorial provided here can aid researchers in the robust design of these widely used experiments and ultimately facilitate the development of new vector control tools. Due to its flexibility, this generic power analysis framework can be customized and extended to inform designs of other vector control SFEs and experimental hut trials. The statistical power analysis framework presented here has already been successfully applied to inform the design of a vector control SFE in one of the semi-field systems in Ifakara, Tanzania.

## Supplementary Information

Additional file 1. List of articles selected for a simple review on the use of power to justify the sample size.

## Data Availability

R tutorials for other SFE scenarios are made freely available as R Markdown files for access on the Zenodo repository (see https://zenodo.org/records/11186504). Some of the parameter values were extracted using mesocosm experimental data from the previous study (see https://www.nature.com/articles/s41598-018–31805-8).

## Abbreviations

VCTs: Vector control tools
ITNs: Insecticide-treated Nets
IRS: Indoor residual spraying
SFEs: Semi-field experiments
SFS: Semi-field Systems
ANOVA: Analysis of variance
GLMs: Generalized linear models
GLMMs: Generalized linear mixed-effect models
PPFa: Pyriproxyfen autodissemination
EV: Estimated variance
HLC: Human landing catch
