# Supplementary material for: A power analysis framework to aid the design of robust semi-field vector control experiments
Source: Malar J. 2025 Jul 18;24:238. doi: 10.1186/s12936-025-05454-y (PMC12275456; doi:10.1186/s12936-025-05454-y)
Supplement: Supplementary file 1 — Additional file 1. List of articles selected for a simple review on the use of power to justify the sample size. [file 12936_2025_5454_MOESM1_ESM.docx]

**Supplementary information**

**A power analysis framework to aid the design of robust semi-field vector control experiments**

Andrea M. Kipingu^1,2,*,^[^ORCID^](https://orcid.org/0000-0002-9063-8651) Dickson W. Lwetoijera^2,^[^ORCID^](https://orcid.org/0000-0003-2544-0451) Kija R. Ng’habi^4^ Samson S. Kiware^2,3,^[^ORCID^](https://orcid.org/0000-0001-7252-520X) Mafalda Viana^1,§,^[^ORCID^](https://orcid.org/0000-0001-5975-6505) Paul C. D. Johnson^1,§,*,^[^ORCID^](https://orcid.org/0000-0001-6663-7520)

**^*^Corresponding authors:** [akipingu@ihi.or.tz](mailto:akipingu@ihi.or.tz) **&** [paul.johnson@glasgow.ac.uk](mailto:paul.johnson@glasgow.ac.uk)

**^§^Joint senior authorship**

**List of affiliations**

1. School of Biodiversity, One Health and Veterinary Medicine, University of Glasgow, Graham Kerr Building, Glasgow G12 8QQ, United Kingdom
2. Department of Environmental Health & Ecological Sciences, Ifakara Health Institute, P.O. Box 78 373, Dar es Salaam, Tanzania
3. The Pan-African Mosquito Control Association, KEMRI Headquarters, Mbagathi Road, Nairobi, Nairobi 54840-00200, Kenya
4. Mbeya College of Health and Allied Sciences, University of Dar es Salaam, P. O. Box 608, Mbeya, Tanzania

**List of email addresses:**

1. Andrea M. Kipingu^1,2,*,^[^ORCID^](https://orcid.org/0000-0002-9063-8651): [akipingu@ihi.or.tz](mailto:akipingu@ihi.or.tz) ([andreakipingu@gmail.com](mailto:andreakipingu@gmail.com))
2. Dickson W. Lwatoejera^2,^[^ORCID^](https://orcid.org/0000-0003-2544-0451): [dwilson@ihi.or.tz](mailto:dwilson@ihi.or.tz)
3. Kija R. Ng’habi^4^: [ndyaisin@gmail.com](mailto:ndyaisin@gmail.com)
4. Samson S. Kiware^2,3,^[^ORCID^](https://orcid.org/0000-0001-7252-520X): [skiware@ihi.or.tz](mailto:skiware@ihi.or.tz)
5. Mafalda Viana^1,§,^[^ORCID^](https://orcid.org/0000-0001-5975-6505): [mafalda.viana@glasgow.ac.uk](mailto:mafalda.viana@glasgow.ac.uk)
6. Paul C. D. Johnson^1,§,*,^[^ORCID^](https://orcid.org/0000-0001-6663-7520): [paul.johnson@glasgow.ac.uk](mailto:paul.johnson@glasgow.ac.uk)

List of selected articles for review.

Malaria Journal articles

Use of power (26%) and no use of power (74%)

1. Vajda, É.A., Saeung, M., Ross, A. *et al.* A semi-field evaluation in Thailand of the use of human landing catches (HLC) versus human-baited double net trap (HDN) for assessing the impact of a volatile pyrethroid spatial repellent and pyrethroid-treated clothing on *Anopheles minimus* landing. *Malar J* **22**, 202 (2023). <https://doi.org/10.1186/s12936-023-04619-x>
2. Muyaga, L.L., Meza, F.C., Kahamba, N.F. *et al.* Effects of vegetation densities on the performance of attractive targeted sugar baits (ATSBs) for malaria vector control: a semi-field study. *Malar J* **22**, 190 (2023). <https://doi.org/10.1186/s12936-023-04625-z>
3. Chanda, J., Wagman, J., Chanda, B. *et al.* Feeding rates of malaria vectors from a prototype attractive sugar bait station in Western Province, Zambia: results of an entomological validation study. *Malar J* **22**, 70 (2023). <https://doi.org/10.1186/s12936-023-04491-9>
4. Gleave, K., Guy, A., Mechan, F. *et al.* Impacts of dual active-ingredient bed nets on the behavioural responses of pyrethroid resistant *Anopheles gambiae* determined by room-scale infrared video tracking. *Malar J* **22**, 132 (2023). <https://doi.org/10.1186/s12936-023-04548-9>
5. Swai, J.K., Kibondo, U.A., Ntabaliba, W.S. *et al.* CDC light traps underestimate the protective efficacy of an indoor spatial repellent against bites from wild *Anopheles arabiensis* mosquitoes in Tanzania. *Malar J* **22**, 141 (2023). <https://doi.org/10.1186/s12936-023-04568-5>
6. Mmbando, A.S., Mponzi, W.P., Ngowo, H.S. *et al.* Small-scale field evaluation of transfluthrin-treated eave ribbons and sandals for the control of malaria vectors in rural Tanzania. *Malar J* **22**, 43 (2023). <https://doi.org/10.1186/s12936-023-04476-8>
7. Yohana, R., Chisulumi, P.S., Kidima, W. *et al.* Anti-mosquito properties of *Pelargonium roseum* (Geraniaceae) and *Juniperus virginiana* (Cupressaceae) essential oils against dominant malaria vectors in Africa. *Malar J* **21**, 219 (2022). <https://doi.org/10.1186/s12936-022-04220-8>
8. Govoetchan, R., Fongnikin, A., Syme, T. *et al.* VECTRON™ T500, a new broflanilide insecticide for indoor residual spraying, provides prolonged control of pyrethroid-resistant malaria vectors. *Malar J* **21**, 324 (2022). <https://doi.org/10.1186/s12936-022-04336-x>
9. Mmbando, A.S., Bradley, J., Kazimbaya, D. *et al.* The effect of light and ventilation on house entry by *Anopheles arabiensis* sampled using light traps in Tanzania: an experimental hut study. *Malar J* **21**, 36 (2022). <https://doi.org/10.1186/s12936-022-04063-3>
10. Tambwe, M.M., Moore, S., Hofer, L. *et al.* Transfluthrin eave-positioned targeted insecticide (EPTI) reduces human landing rate (HLR) of pyrethroid resistant and susceptible malaria vectors in a semi-field simulated peridomestic space. *Malar J* **20**, 357 (2021). <https://doi.org/10.1186/s12936-021-03880-2>
11. Kaindoa, E.W., Mmbando, A.S., Shirima, R. *et al.* Insecticide-treated eave ribbons for malaria vector control in low-income communities. *Malar J* **20**, 415 (2021). <https://doi.org/10.1186/s12936-021-03945-2>
12. Mbuba, E., Odufuwa, O.G., Tenywa, F.C. *et al.* Single blinded semi-field evaluation of MAÏA^®^ topical repellent ointment compared to unformulated 20% DEET against *Anopheles gambiae*, *Anopheles arabiensis* and *Aedes aegypti* in Tanzania. *Malar J* **20**, 12 (2021). <https://doi.org/10.1186/s12936-020-03461-9>
13. Osoro, J.K., Machani, M.G., Ochomo, E. *et al.* Insecticide resistance exerts significant fitness costs in immature stages of *Anopheles gambiae* in western Kenya. *Malar J* **20**, 259 (2021). <https://doi.org/10.1186/s12936-021-03798-9>
14. Nignan, C., Niang, A., Maïga, H. *et al.* Comparison of swarming, mating performance and longevity of males *Anopheles coluzzii* between individuals fed with different natural fruit juices in laboratory and semi-field conditions. *Malar J* **19**, 173 (2020). <https://doi.org/10.1186/s12936-020-03248-y>
15. Martin, N.J., Nam, V.S., Lover, A.A. *et al.* The impact of transfluthrin on the spatial repellency of the primary malaria mosquito vectors in Vietnam: *Anopheles dirus* and *Anopheles minimus*. *Malar J* **19**, 9 (2020). <https://doi.org/10.1186/s12936-019-3092-4>
16. Sangoro, O.P., Gavana, T., Finda, M. *et al.* Evaluation of personal protection afforded by repellent-treated sandals against mosquito bites in south-eastern Tanzania. *Malar J* **19**, 148 (2020). <https://doi.org/10.1186/s12936-020-03215-7>
17. Cribellier, A., Spitzen, J., Fairbairn, H. *et al.* Lure, retain, and catch malaria mosquitoes. How heat and humidity improve odour-baited trap performance. *Malar J* **19**, 357 (2020). <https://doi.org/10.1186/s12936-020-03403-5>
18. Kemibala, E.E., Mafra-Neto, A., Dekker, T. *et al.* A zooprophylaxis strategy using L-lactic acid (Abate) to divert host-seeking malaria vectors from human host to treated non-host animals. *Malar J* **19**, 52 (2020). <https://doi.org/10.1186/s12936-020-3136-9>
19. Musa, J.J., Moore, S., Moore, J. *et al.* Long-lasting insecticidal nets retain bio-efficacy after 5 years of storage: implications for malaria control programmes. *Malar J* **19**, 110 (2020). <https://doi.org/10.1186/s12936-020-03183-y>

**Parasites & Vectors articles**

Use of power (32%) and no use of power (68%)

1. Zahouli, J.Z.B., Dibo, JD., Diakaridia, F. *et al.* Semi-field evaluation of the space spray efficacy of Fludora Co-Max EW against wild insecticide-resistant *Aedes aegypti* and *Culex quinquefasciatus* mosquito populations from Abidjan, Côte d’Ivoire. *Parasites Vectors* **16**, 47 (2023). <https://doi.org/10.1186/s13071-022-05572-5>
2. Maasayi, M.S., Machange, J.J., Kamande, D.S. *et al.* The MTego trap: a potential tool for monitoring malaria and arbovirus vectors. *Parasites Vectors* **16**, 212 (2023). <https://doi.org/10.1186/s13071-023-05835-9>
3. Tia, I.Z., Barreaux, A.M.G., Oumbouke, W.A. *et al.* Efficacy of a ‘lethal house lure’ against *Culex quinquefasciatus* from Bouaké city, Côte d’Ivoire. *Parasites Vectors* **16**, 300 (2023). <https://doi.org/10.1186/s13071-023-05883-1>
4. Tambwe, M.M., Kibondo, U.A., Odufuwa, O.G. *et al.* Human landing catches provide a useful measure of protective efficacy for the evaluation of volatile pyrethroid spatial repellents. *Parasites Vectors* **16**, 90 (2023). <https://doi.org/10.1186/s13071-023-05685-5>
5. Sarwar, M.S., Jahan, N., Ali, A. *et al.* Establishment of *Wolbachia* infection in *Aedes aegypti* from Pakistan via embryonic microinjection and semi-field evaluation of general fitness of resultant mosquito population. *Parasites Vectors* **15**, 191 (2022). <https://doi.org/10.1186/s13071-022-05317-4>
6. Njoroge, M.M., Hiscox, A., Saddler, A. *et al.* Less is more: repellent-treated fabric strips as a substitute for full screening of open eave gaps for indoor and outdoor protection from malaria mosquito bites. *Parasites Vectors* **15**, 259 (2022). <https://doi.org/10.1186/s13071-022-05384-7>
7. Pauly, I., Jakoby, O. & Becker, N. Efficacy of native cyclopoid copepods in biological vector control with regard to their predatory behavior against the Asian tiger mosquito, *Aedes albopictus*. *Parasites Vectors* **15**, 351 (2022). <https://doi.org/10.1186/s13071-022-05460-y>
8. Mbewe, N.J., Rowland, M.W., Snetselaar, J. *et al.* Efficacy of bednets with dual insecticide-treated netting (Interceptor® G2) on side and roof panels against *Anopheles arabiensis* in north-eastern Tanzania. *Parasites Vectors* **15**, 326 (2022). <https://doi.org/10.1186/s13071-022-05454-w>
9. Kibondo, U.A., Odufuwa, O.G., Ngonyani, S.H. *et al.* Influence of testing modality on bioefficacy for the evaluation of Interceptor^®^ G2 mosquito nets to combat malaria mosquitoes in Tanzania. *Parasites Vectors* **15**, 124 (2022). <https://doi.org/10.1186/s13071-022-05207-9>
10. Tambwe, M.M., Saddler, A., Kibondo, U.A. *et al.* Semi-field evaluation of the exposure-free mosquito electrocuting trap and BG-Sentinel trap as an alternative to the human landing catch for measuring the efficacy of transfluthrin emanators against *Aedes aegypti*. *Parasites Vectors* **14**, 265 (2021). <https://doi.org/10.1186/s13071-021-04754-x>
11. Bokore, G.E., Svenberg, L., Tamre, R. *et al.* Grass-like plants release general volatile cues attractive for gravid *Anopheles gambiae* sensu stricto mosquitoes. *Parasites Vectors* **14**, 552 (2021). <https://doi.org/10.1186/s13071-021-04939-4>
12. Njoroge, M.M., Fillinger, U., Saddler, A. *et al.* Evaluating putative repellent ‘push’ and attractive ‘pull’ components for manipulating the odour orientation of host-seeking malaria vectors in the peri-domestic space. *Parasites Vectors* **14**, 42 (2021). <https://doi.org/10.1186/s13071-020-04556-7>
13. Nambunga, I.H., Msugupakulya, B.J., Hape, E.E. *et al.* Wild populations of malaria vectors can mate both inside and outside human dwellings. *Parasites Vectors* **14**, 514 (2021). <https://doi.org/10.1186/s13071-021-04989-8>
14. Tambwe, M.M., Moore, S.J., Chilumba, H. *et al.* Semi-field evaluation of freestanding transfluthrin passive emanators and the BG sentinel trap as a “push-pull control strategy” against *Aedes aegypti* mosquitoes. *Parasites Vectors* **13**, 392 (2020). <https://doi.org/10.1186/s13071-020-04263-3>
15. Sippy, R., Rivera, G.E., Sanchez, V. *et al.* Ingested insecticide to control *Aedes aegypti*: developing a novel dried attractive toxic sugar bait device for intra-domiciliary control.*Parasites Vectors* **13**, 78 (2020). <https://doi.org/10.1186/s13071-020-3930-9>
16. Hughes, A., Lissenden, N., Viana, M. *et al.* *Anopheles gambiae* populations from Burkina Faso show minimal delayed mortality after exposure to insecticide-treated nets. *Parasites Vectors* **13**, 17 (2020). <https://doi.org/10.1186/s13071-019-3872-2>
17. Kathet, S., Sudi, W., Mwingira, V. *et al.* Efficacy of 3D screens for sustainable mosquito control: a semi-field experimental hut evaluation in northeastern Tanzania. *Parasites Vectors* **16**, 417 (2023). <https://doi.org/10.1186/s13071-023-06032-4>
18. Cozzarolo, CS., Pigeault, R., Isaïa, J. *et al.* Experiment in semi-natural conditions did not confirm the influence of malaria infection on bird attractiveness to mosquitoes. *Parasites Vectors* **15**, 187 (2022). <https://doi.org/10.1186/s13071-022-05292-w>
19. Fongnikin, A., Houeto, N., Agbevo, A. *et al.* Efficacy of Fludora® Fusion (a mixture of deltamethrin and clothianidin) for indoor residual spraying against pyrethroid-resistant malaria vectors: laboratory and experimental hut evaluation. *Parasites Vectors* **13**, 466 (2020). https://doi.org/10.1186/s13071-020-04341-6

Total in all journals combined: Use of power (29%) and no use of power (71%)
